# Visualisation of hypertension: A non-randomised pilot study to explore the feasibility of a Community Pharmacy-based intervention to support medication adherence (Hi-BP)

**DOI:** 10.1371/journal.pone.0339871

**Published:** 2026-01-02

**Authors:** Sarah L. Brown, Barry J. McDonnell, David McRae, Paul Angel, Imtiaz Khan, Rhiannon Phillips, Britt Hallingberg, Delyth H. James

**Affiliations:** 1 School of Sport and Health Sciences, Cardiff Metropolitan University, Cardiff, Wales, United Kingdom; 2 Cwm Taf Morgannwg University Health Board, Wales, United Kingdom; 3 School of Technologies, Cardiff Metropolitan University, Cardiff, Wales, United Kingdom; 4 School of Medicine, Department of Pharmacy, Swansea University, Wales, United Kingdom; The University of Lahore, PAKISTAN

## Abstract

Using visualisation to conceptualise a chronic condition can encourage accurate illness beliefs and support treatment adherence. Hi-BP is a digital visual intervention to support adherence to antihypertensive medication, co-produced with patients. The aim of this study was to investigate the feasibility and acceptability of Hi-BP and explore the preliminary direction of effects on illness and treatment beliefs, medication adherence and blood pressure (BP). A two-phased mixed-methods non-randomised feasibility study was conducted from April 2021 to March 2022 in eight community pharmacies across one Health Board in South-East Wales, UK. Hi-BP was delivered as a single researcher-led consultation to 69 patients in Phase 1 and by pharmacists to three patients in Phase 2. Feasibility was determined using predefined criteria, with acceptability explored qualitatively using semi-structured interviews. Quantitative outcome measures (*illness perceptions, medication beliefs, medication-adherence, prescription dispensing and collection data, BP)* were recorded at baseline and immediately post-intervention.Follow-up outcome measures were collected at two-weeks (*medication-adherence*) and three-months (*all baseline measures*). Hi-BP met feasibility criteria for pharmacist recruitment in both phases, and patient recruitment in Phase 1, but not Phase 2. Hi-BP was acceptable to the sub-sample of 15 patient participants interviewed in Phase 1; insufficient data were available to determine patient acceptability at Phase 2. Hi-BP was acceptable to pharmacists in Phase 1 and partially acceptable at Phase 2, due to competing demands on time for intervention delivery. All outcome measures were considered feasible for use, though a ceiling effect was noted for medication adherence. A potentially positive directional effect was found for illness perceptions (X^2^(2)=10.83,n=54,p=0.004), medication beliefs (BMQ-Necessity (X^2^(2)=11.71,n=54,p=0.003) and BP (Systolic BP Z=-3.91,n=51,p=<0.001) but not for medication adherence (MARS-5 X^2^(2)= 2.4,n=45,p=0.299). In the Community Pharmacy setting, Hi-BP was well-accepted and has the potential for significant reductions in BP; however, further research is needed to explore pharmacist capacity to support implementation.

## Introduction

Hypertension (high blood pressure) is an asymptomatic chronic condition, the greatest single risk factor contributing to worldwide mortality [1], strongly associated with the incidence of cardiovascular disease [2,3]. Hypertension is prevalent worldwide, with around a third of adults affected, and is associated with eleven million deaths per year [4,5]. While hypertension management has improved over the years, even in countries with high rates of treatment less than 70% of those diagnosed achieve appropriate control [6]. Despite evidence that adherence to antihypertensive medication improves cardiovascular outcomes [7–9], non-adherence to treatment is prevalent, reported as between 27–40% [10] and highlighted as a global issue by the World Health Organisation [11]. Non-adherence has multiple determinants: social and economic, health system, condition-, therapy-, and patient-related factors [11,12].

A variety of interventions have been explored to improve medication adherence in a range of chronic conditions and settings. However, no strategy has been proven effective in all settings [13] in addressing both adherence and clinical outcomes [14]. The most successful interventions are multifaceted, with those facilitated by pharmacists or allied health professionals being the most effective [14]. Research using psychological theories has been conducted to explain and predict health-related behaviour, such as medication adherence. Patient perceptions of illness and beliefs about treatment have been identified as significant determinants of non-adherence [15,16] and shown to influence adherence to antihypertensive treatment [17–19]. The asymptomatic nature of hypertension appears to influence non-adherence, as the perceived benefits of treatment are undetectable to patients [20,21].

The use of visual materials is one method of conceptualising an abstract condition such as hypertension. Use of visualisation to conceptualise a chronic condition has been shown to encourage the development of accurate illness beliefs, and support treatment adherence [22–24]. Furthermore, use of pictures to accompany written text or spoken narrative has been shown to increase attention to and recall of health messaging [25]. Visuals can also aid in the communication of personalised disease risk [26].

**Fig 1:**
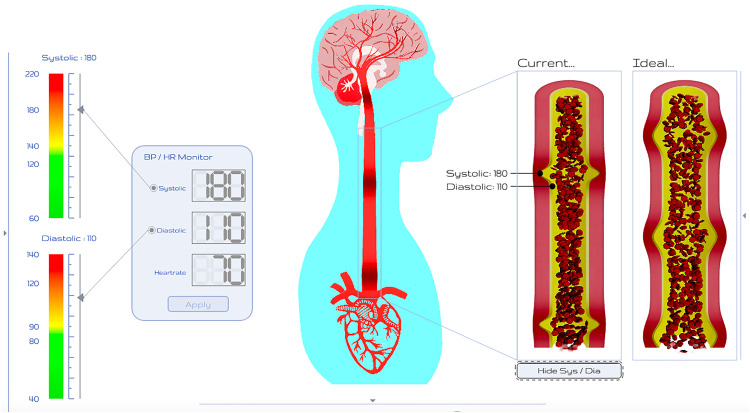
Visual of Hi-BP platform.

Hi-BP is a digital visual intervention, designed for use within a healthcare professional-facilitated consultation (Fig 1). The primary purpose of Hi-BP was to enable the visualisation and conceptualisation of hypertension to help patients make the connection between their measured BP and the impact of this on their physiology and risk of cardiovascular events. In this way, Hi-BP improves public and patient understanding of hypertension, by addressing illness perceptions and beliefs about the perceived necessity of treatment to support antihypertensive medication adherence. Hi-BP was developed by a multi-disciplinary team to support medication adherence using six behaviour change techniques, as described by the Behaviour Change Techniques Taxonomy v1 [27]: *2.6 Biofeedback, 2.7 Feedback on outcome(s) of behaviour, 5.1 Information about health consequences, 5.2 Salience of consequences, 9.1 Credible source* and *9.3, Comparative imagining of future outcomes*. It was iteratively developed using the Medical Research Council (MRC) framework for complex interventions [28] as a guide to facilitate and incorporate stakeholder and end-user feedback. Two psychological theories underpinned the intervention: the Common-Sense Model of Self-Regulation (CSM) [29–31] and Necessity-Concerns Framework (NCF) [32].

The primary aim of this pilot study was to investigate the feasibility and acceptability of the Hi-BP intervention and its delivery in a community pharmacy-based setting. The feasibility of delivering Hi-BP in a CP setting and its acceptability to pharmacists and patients were evaluated, including screening for participant eligibility and recruitment. The pilot study also aimed to explore the acceptability of the outcome measures and initial direction of effect of the intervention on illness and treatment beliefs, medication adherence and blood pressure.

## Materials and methods

### Study overview

A two-phased mixed methods non-randomised pilot feasibility study was conducted in community pharmacies (CPs) in South-East Wales between April 2021 and March 2022. Ethical approval was granted by NHS Research Ethics Committee (Wales REC 5 Ref: 20/WA/0280) and Cardiff Metropolitan University, Cardiff School of Sport & Health Sciences, Ethics Committee (Ref: PGR-3806).

**Fig 2:**
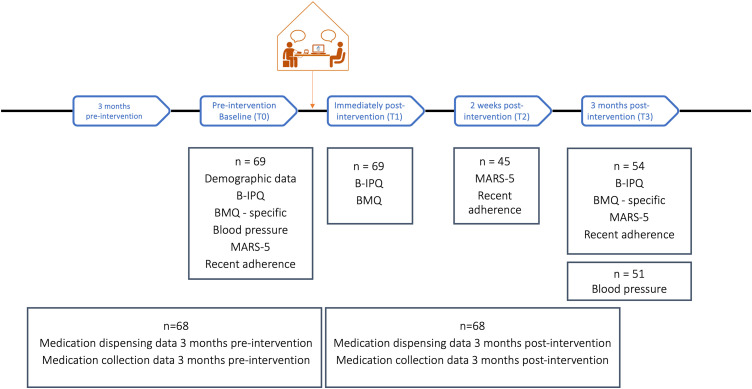
Quantitative measures collected at each timepoint.

The Hi-BP intervention was delivered as a single consultation by a researcher pharmacist (SB) in Phase 1 and by the usual pharmacists in Phase 2. A two-phase approach was employed to assess aspects of the feasibility and acceptability of Hi-BP in the intended setting (community pharmacy), with minimal burden on pharmacists and pharmacy staff. In line with the MRC framework for complex interventions, had any major issues with recruitment or intervention delivery been identified at this stage, amendments would have been made before proceeding to Phase 2. Quantitative outcome measures were recorded at baseline and immediately post-intervention. Follow-up outcome measures were collected at 2-weeks (medication adherence only) and at 3-months (all outcomes assessed) (Fig 2). A detailed description of the intervention content and associated facilitator training can be found in Supplementary Material S1 File.

### Feasibility assessment

Feasibility assessment was undertaken using *a priori* criteria considering both process feasibility and acceptability of the Hi-BP intervention for patients and pharmacists. Feasibility of participant recruitment was considered in terms of appropriateness of recruitment, i.e., the percentage of participants who met the eligibility criteria, and participant numbers compared to recruitment target. Retention of patient participants was determined by the proportion of patients who provided follow-up data at 3-months.

### Process feasibility

#### Phase 1: recruitment of pharmacy sites and patient sampling.

All 34 community pharmacies from a multiple-pharmacy chain in one geographical area of South-East Wales were invited to participate. Five pharmacies consented to take part, with one participating pharmacist in each site. Recruitment from a single pharmacy chain ensured uniformity in standard operating procedures for reordering repeat prescriptions, providing consistency in capturing adherence data. Prescription dispensing ranged from 6,000–20,000 items per month and all participating pharmacies also provided a broad range of additional services. The patient recruitment target was 100 (20 from each study site). As this was a pilot study, a power calculation was not deemed necessary [33], therefore sample size was informed by other feasibility studies for pharmacy-based medication-related interventions [34–36].

Eligible patients were over 18 years old, with an existing diagnosis of hypertension, prescribed at least one antihypertensive medication for a minimum of three months, and using one of the study pharmacies to pick up their prescriptions. Individuals were not eligible if they had a medication compliance aid prepared by the pharmacy or a visual impairment that prevented them from viewing a computer or electronic tablet screen.

Hypertensive patients were recruited between June and August 2021 by the participating pharmacy either by: (1) spontaneous recruitment, through identification of suitable patients when the individual was collecting a prescription or (2) direct recruitment, where eligible individuals were identified through pharmacy records and contacted directly, via telephone, mailshot or using an informational leaflet attached to their medication bag.

#### Phase 2: recruitment of pharmacists and patients.

All pharmacists employed by the pharmacy chain in Phase 1 were invited to participate via email. Recruitment from the same chain provided consistency between phases. The recruitment target of five participating pharmacies was deemed appropriate, to enable a variety of locations and business sizes, following guidelines for qualitative aspects of feasibility testing [37]. Patient recruitment conducted between November 2021 and January 2022 followed the same approach as Phase 1.

Retention was determined by the number of participants providing data at each timepoint.

### Acceptability

Acceptability of the intervention for both patients and pharmacists was explored qualitatively through semi-structured interviews (Phase 1 – a purposive sample of patients; Phase 2 – all pharmacists) and written feedback (Phase 1 – pharmacy sites only). Purposive sampling of patients was based on the pharmacy site and change in outcome scores over time.

### Direction of effect: quantitative outcome measures

#### Illness perceptions and medication beliefs.

Outcome measures were recorded at baseline (T0), immediately post-intervention (T1) and at three-month follow-up (T3), for illness beliefs, medication adherence and BP, to investigate the preliminary direction of effect and further inform the design of a future evaluative trial. The appropriateness of outcome measures was assessed through the percentage of self-report questionnaires with missing data. Illness and medication beliefs were captured using the Brief Illness Perception Questionnaire (B-IPQ) [38] modified for hypertension and the Beliefs about Medicines Questionnaire-Specific Subscale (BMQ-S) [39] adapted for anti-hypertensive medication.

#### Medication adherence.

The implementation phase of medication adherence was studied, i.e., the extent to which the patient’s medication-taking corresponds to the agreed dosing regimen [40], using both self-report questionnaires and pharmacy data. The MARS-5 questionnaire [41] and a three-item measure of recent adherence capturing medication taken over the previous two weeks were used. The date of antihypertensive medication dispensing and the date the prescription was collected from the pharmacy, where data were available, were used to calculate the Medication Possession Ratio (MPR) for the three months pre-intervention and post-intervention. MPR was calculated using the formula: MPR = number of days’ supply in the refill interval/number of days in the refill interval.

#### Blood pressure.

BP was measured three times in the seated position, with the average of the second and third reading used for analysis, according to BIHS guidelines [42]. Measurements were taken at baseline (T0) and three-month follow-up (T3) using a semi-automated oscillometric blood pressure device (Omron M3™). Depending on patient preference, a home BP reading taken within one week of the consultation could also be used in preference to an in-consultation measurement. Demographic information including age, gender, education, number of antihypertensive medications and number of other long-term medications prescribed was recorded.

### Analysis

Qualitative data were analysed using Template Analysis [43], using *a priori* themes where data were mapped to the seven constructs of the Theoretical Framework of Acceptability [44]. Other inductively derived themes were added to the thematic template as they were constructed.

Statistical analyses were undertaken using Statistical Package for the Social Sciences (SPSS) v27. Non-parametric statistical tests were used to compare scores for illness perceptions and medication beliefs, adherence and BP over time, due to the presence of outlying scores and a strong positive skew for some measures. Data are therefore reported as median (Md) and interquartile range (IQR).

## Results

### Process feasibility

Overall, pharmacist recruitment was considered feasible, and the recruitment target was met, with pharmacists from a total of eight pharmacy branches participating. Two pharmacies had pharmacists participating in both phases, three pharmacies had pharmacists participating in phase one only, and a further three participated in Phase 2 only. The recruitment target for patient participants was met in Phase 1, with all pharmacies recruiting at least one patient per week throughout the study period. All recruited patients met the eligibility criteria, meaning the screening of appropriate patient participants was also feasible in this setting. The patient attrition rate for Phase 1 was 21.7%, which is within the 28% feasibility threshold. However, Phase 2 patient recruitment targets were not met, with only three patients recruited overall. Due to insufficient data in Phase 2, it was not possible to assess whether the intervention methodology had been delivered as intended by pharmacist facilitators. Further details for each phase are provided below.

### Phase 1

#### Community pharmacy (CP) and patient recruitment.

The recruitment target for CP and patient participants was met, with all pharmacies recruiting at least one patient per week throughout the study period. One of the pharmacies chose to contact eligible patients via mail as part of their direct recruitment. Recruitment letters were sent to the first 65 eligible participants identified from the Pharmacy Patient Medication Record, from which four participants responded and were recruited to the study. It is not known how many individuals were invited spontaneously, or how many of the informational leaflets were given to patients, therefore it is not possible to calculate a response rate for these recruitment methods. In total, 88 individuals expressed an interest in taking part and 69 (78%) provided their informed consent. Reasons for non-participation were mainly related to availability to attend an appointment within the weekday opening hours of the pharmacy (Monday to Friday between 9am to 6 or 7 pm). Recruitment varied from eight patients in one pharmacy to 17 (median = 14).

**Fig 3:**
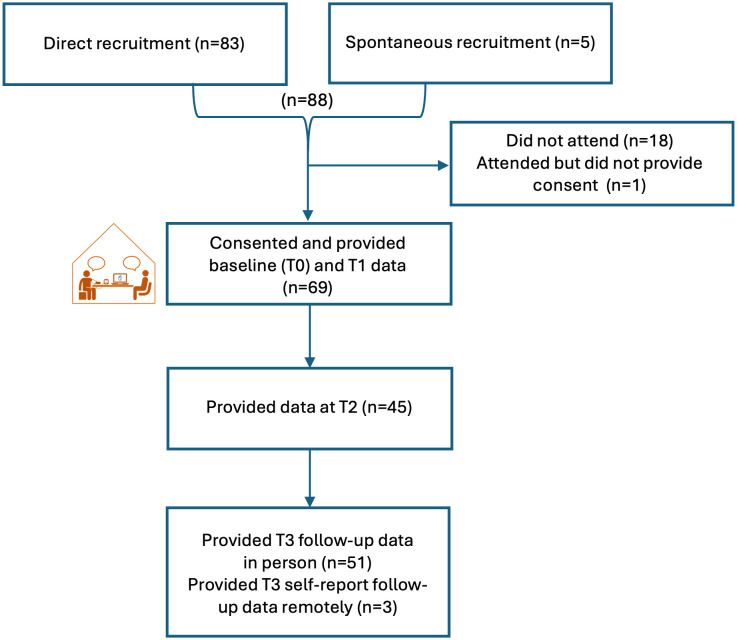
Patient participant flow through Phase 1.

Patient participants’ age ranged from 49 to 84 years (mean 68.9, SD = 6.85). Thirty-eight (55.1%) were male, and 68/69 (98.6%) were white, with one participant reporting mixed ethnicity. Almost half (n = 33; 47.8%) were educated to the end of compulsory education (16-years old in the UK). The mean number of years since their hypertension diagnosis was 14.67 (range 0.5–45 years; SD = 9.22), with most prescribed one or two antihypertensive medications (range 1–5; median = 2). The majority of patients (64; 92.8%) were also prescribed medications for other long-term conditions (range 0–11; median = 3). All pharmacies were located in geographical areas ranked in the most deprived 60% of areas in Wales, i.e., within the lowest three deprivation quintiles [45]. All patients attending consultation one provided both baseline (T0) and post-intervention (T1) data. Of these patients, 45 (65%) provided adherence self-report data two-weeks post-intervention (T2). In total 78% provided three-month follow-up (T3) data; fifty-one (74%) visited the pharmacy for this and three (4%) provided data remotely, meaning BP data was not available for these (Fig 3).

### Phase 2

#### Pharmacist recruitment.

Six pharmacist participants engaged with Phase 2 of the research across five pharmacy branches. Three pharmacists from Phase 1 declined to participate in Phase 2, and a further four were recruited. Since two of the pharmacists worked in the same branch, the intervention was delivered across five branches in Phase 2. Reasons provided for those three branches that did not continue to Phase 2 related to workload (e.g., prioritising delivery of remunerated services) and lack of staff continuity (e.g., pharmacists leaving the pharmacy).

#### Patient recruitment.

Three patients were recruited by two pharmacists in two different branches between November 2021 and March 2022. The remaining four pharmacists did not recruit any participants; therefore, the recruitment target was not met. Self-report adherence data at two-weeks post-intervention were returned for two patients, and no data were returned for the three-month follow-up consultation.

### Acceptability: qualitative data (Phases 1 and 2)

A sub-sample of fifteen Phase 1 patient participants provided feedback during one-to-one interviews and three pharmacists participating in Phase 1 provided their views via email. All six Phase 2 pharmacists shared their experiences via online or in-person semi-structured interviews.

**Fig 4:**
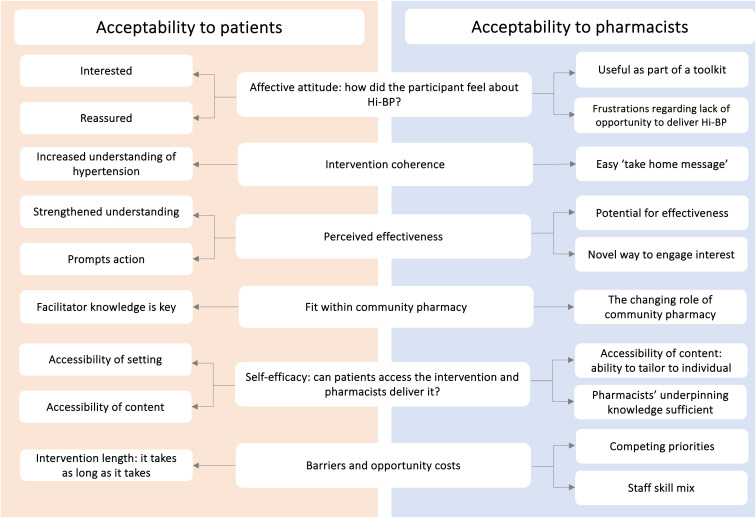
Qualitative themes derived from the Theoretical Framework of Acceptability.

Six themes were constructed from the data across both phases (Fig 4). These were 1) ‘Affective attitude: how did the participant feel about Hi-BP’, 2) ‘Intervention coherence: did participants understand Hi-BP’, 3) ‘Perceived effectiveness: how does Hi-BP achieve its aim?’, 4) ‘Fit within community pharmacy: does Hi-BP have a place within current practice?’, 5) ‘Self-efficacy: can patients access the intervention and pharmacists deliver it? and 6) ‘Barriers and opportunity costs: what has to be given up to deliver Hi-BP?’

#### Theme 1: affective attitude.

Patients described being interested in the intervention and those with maximal adherence were reassured that their medication-taking had produced the desired effect. Pharmacists were enthusiastic towards the intervention, with those in Phase 1 finding recruitment straightforward and good acceptance to participate. While Phase 2 pharmacists were keen to deliver Hi-BP consultations, there were frustrations around time-sensitive and remunerated tasks taking priority.

#### Theme 2: intervention coherence.

Patients felt Hi-BP helped them make sense of their hypertension citing it as particularly helpful for newly diagnosed patients or those with a longstanding diagnosis to maintain the perceived importance of treatment. Pharmacists quickly understood the aim and need for Hi-BP, providing an easy ‘take-home message’ for patients, illustrating that hypertension was a condition to take seriously but to reassure them that it could be effectively managed.

#### Theme 3: perceived effectiveness.

Patients felt that Hi-BP strengthened their understanding of hypertension and provided a clear rationale for antihypertensive treatment, and reassurance regarding the effectiveness of the medication. They also believed that this visual prompt would encourage other people to make positive changes to address their hypertension. Pharmacists could see the potential for intervention effectiveness, particularly with newly diagnosed individuals, older people, or those taking multiple antihypertensives. It was suggested that the visuals would provide an appealing, novel way of re-engaging patients’ interest in their condition.

#### Theme 4: fit within community pharmacy.

Patients believed that the CP was a good place to host the intervention since the healthcare setting provided credibility and good access. The attributes of the person delivering the Hi-BP consultation were considered key to its success. Patient opinion was divided on whether a healthcare background was necessary, with some suggesting that an in-depth understanding of the intervention and effective communication skills were sufficient. It was suggested that suitably trained healthcare staff could deliver the intervention.

Pharmacists saw medicines adherence support as a core part of their responsibilities and highlighted the evolving role of community pharmacists in the UK towards being more clinically focused. However, they described taking an ad-hoc approach to supporting patients with medicines management since the decommissioning of a remunerated service in 2020 in Wales, which led to a perception of reduced emphasis for this type of activity in a CP-setting. Some pharmacists suggested that chronic medicine management was more suited to General Practice (GP)-based pharmacists than CP.

#### Theme 5: Self-efficacy: can patients access the intervention and pharmacists deliver it?.

Patients felt confident that the intervention setting and content of the intervention itself were appropriate to achieve the intended goal. Accessibility was key, in terms of ease of access to the CP setting, which was considered to be more accessible than a GP surgery, highlighting the importance of feeling relaxed in a familiar setting during the consultation. Patients believed that the utility of visuals and understandable language was a strength of the intervention content which would also be suitable for those with lower educational levels. The in-person facilitated conversation was welcomed over a self-directed digital application or remote consultation.

Pharmacists also thought that the CP setting was a convenient location to deliver Hi-BP as CP is now seen as a first port of call for many patients, with increasing volume of people approaching them for advice. Pharmacists also believed the content was accessible for patients, providing agency to tailor the conversation to the patient’s needs. Participating pharmacists felt confident that their understanding of hypertension was sufficient to facilitate the intervention, with some choosing to refresh their knowledge of clinical guidelines beforehand.

#### Theme 6: barriers and opportunity costs: what has to be given up to deliver Hi-BP?.

Patients did not offer any direct barriers or opportunity costs when asked. Whilst they acknowledged that the consultation was longer than previously experienced, they welcomed the opportunity to discuss and understand their hypertension. In contrast, lack of time and competing priorities were mentioned by all pharmacists as a potential barrier to delivering the intervention. Whilst they described wanting to provide new pharmacy services such as this, the daily balance between this and prescription dispensing made it a challenge, and priority often given to remunerated activities and managing acute conditions. Their workload was perceived to have increased in recent years, with changes to how and where patients access primary care being hastened by the COVID-19 pandemic. Pharmacists noted that the nature of the skill mix in the pharmacy team also impacted their workload, making it difficult to delegate tasks in order for them to engage with new patient-facing services such as this. Two pharmacists who had consented to take part in Phase 2 subsequently realised these challenges which led to no attempt being to recruit patients. For indicative quotes, see Supplementary Material S2 File.

In summary, Phase 1 demonstrates the acceptability of Hi-BP as a researcher-facilitated intervention, however, Phase 2 highlighted issues at the time with the feasibility of implementation, as a pharmacist-facilitated intervention, in this setting. Table 1 summarises the feasibility and acceptability indicators of Hi-BP and its delivery in a community pharmacy-based setting across Phases 1 and 2 of the study.

**Table 1:** Feasibility and acceptability indicators for Hi-BP delivered in a community pharmacy setting.

Feasibility criteria	Measures used	Criteria met if:	Feasibility criteria met:	
			Phase 1	Phase 2
1a. Feasibility of recruiting sufficient pharmacists. 1b. Feasibility of recruiting sufficient participants.	Number of pharmacists recruited as a percentage of the target. Number of participants recruited/week.	80% of the desired number of pharmacists were recruited (target sample size = 5 pharmacists). Participating pharmacies able to recruit ≥ 1 participant/week during recruitment period (Phase 1). Participating pharmacies able to recruit ≥ 1 participant/fortnight during 10-week recruitment period (Phase 2).	Yes Yes n/a	Yes n/a **No**
2. Feasibility of screening and recruiting suitable participants.	Recruited participants met the eligibility criteria (Phase 1 & 2).	All participants had been prescribed an antihypertensive medication for at least three months prior to consultation as determined by pharmacy medication record data and confirmed by patient.	Yes	Yes
3. Intervention methodology delivered as intended, i.e., the ViSTA-BP tablet application incorporated into the consultation.	ViSTA-BP app accessed during the consultation by pharmacist facilitators (Phase 2). Use of the app explored through recall of pharmacists’ consultation experience during qualitative interviews.	ViSTA-BP app accessed for at least 10 minutes during each consultation.	n/a	Unclear (Insufficient data)
4. Adherence and illness beliefs outcome measures feasible for use.	Missing data within completed self-report questionnaires.	No more than 5% of the B-IPQ, BMQ, MARS-5 and recent adherence questionnaires had missing data.	Yes	Unclear (Insufficient data)
5. ViSTA-BP intervention content acceptable to patients	Issues with acceptability explored through qualitative interviews using the Theoretical Framework of Acceptability. Percentage of participants returning for three-month follow-up	Positive responses provided for: • Affective attitude • Intervention coherence • Perceived effectiveness • Ethicality: fit within community pharmacy • Opportunity costs No more than 28% attrition by three-month follow-up.	Affective attitude: Yes Intervention coherence: yes Perceived effectiveness: Yes Fit within community pharmacy: Yes Opportunity costs: Yes Yes	Unclear (Insufficient data) **No**
6. ViSTA-BP intervention content acceptable to pharmacist facilitators	Issues with acceptability explored through qualitative interviews using the Theoretical Framework of Acceptability	Positive responses provided for: • Affective attitude • Intervention coherence • Perceived effectiveness • Ethicality • Burden	Affective attitude: Yes Intervention coherence: Yes Perceived effectiveness: Yes Fit within community pharmacy: Partial Burden: Yes	Affective attitude: Yes Intervention coherence: Yes Perceived effectiveness: Yes Fit within community pharmacy: Partial Burden: **No**

### Direction of effect: quantitative outcome Measures (Phase 1)

The adherence, illness perceptions and medication beliefs outcome measures were considered feasible for use, as there was no missing data within completed self-report questionnaires in Phase 1.

#### Illness and medication beliefs outcomes.

There was a statistically significant difference in scores across the three timepoints for the total B-IPQ score (X^2^(2)=10.83, n = 54, p = 0.004). Median scores decreased between baseline (32.50, interquartile range [IQR] 24.75–39.00) and T1 (28.50, IQR 24.00–35.50), increasing slightly at T3 (30.00, IQR 25.25–35.00), indicating that participants had a less threatening view of their hypertension post-intervention. There were also significant positive differences for three of the individual B-IPQ constructs: personal control (X^2^(2)= 6.16, n = 54, p = 0.046), treatment control (X^2^ (2)=9.04, n = 54, p = 0.011), and illness coherence (X^2^ (2)=32.35, n = 54, p=<0.001), suggesting that participants had greater illness coherence and a more positive perception of personal control over their hypertension and the utility of their treatment for controlling their hypertension post-intervention.

There was a significant positive difference in BMQ necessity subscale scores across the three timepoints (X^2^(2)=11.71, n = 54, p = 0.003). BMQ concern subscale scores also showed significant differences over time (X^2^(2)=11.71, n = 54, p = 0.003), with concern scores lowering immediately post-intervention and increasing slghtly at three-month follow-up. The necessity-concerns differential (NCD) was calculated over the three timepoints, showing a statistically significant increase (X^2^(2)=9.95, n = 54, p = 0.007). At baseline, 14.5% of participants (10/69) had an NCD of less than zero, indicating that they had greater concerns about their antihypertensives than belief in the necessity of the treatment. At T1 this percentage had dropped to just 1.4%, with only one participant scoring less than zero. At T3 no one had an NCD less than zero, indicating that by three-month follow-up the perceived necessity for antihypertensives was greater than the concerns about taking the medication for all participants.

#### Adherence outcomes.

There was no statistically significant change in MARS-5 scores across the three timepoints (X^2^ (2)= 2.4, n = 45, p = 0.299). The median score at all three timepoints was 25 (out of a maximum of 25), demonstrating that self-reported adherence was consistently high, however, the range of scores indicates there was some improvement over time (baseline Md 25, range 9−25, IQR 24−25; T2 Md 25, range 18−25, IQR 24−25; T3 Md 25, range 20−25, IQR 25−25). A similar pattern was seen with the recent adherence scores, with no statistically significant difference reported across the three timepoints (X^2^(2)=5.89, n = 45, p = 0.053). As with the MARS-5 scores, the range of scores indicates there was a move towards greater adherence over time, with 60% (27/45) reporting 100% adherence at baseline, 73.3% (33/45) 100% adherence two weeks post-intervention and 80% (36/45) at three-month follow-up. Sixty-eight participants had valid data for medication dispensing for the three months pre-intervention and post-intervention. No statistically significant difference was found pre- and post-intervention (Z = −0.76, n = 68, p = 0.455). Medication collection data also showed no significant change in median scores (Z = −1.10, n = 45, p = 0.271).

#### Blood pressure measurements.

There was a statistically significant change in systolic blood pressure (SBP) between baseline and three-month follow-up (Z = −3.91, n = 51, p=<0.001). The median SBP reduced from 148 mmHg (IQR 135 mmHg to 160 mmHg) at baseline, to 133 mmHg (IQR 126 mmHg to 148 mmHg) at three-month follow-up. A similar result was observed for diastolic blood pressure (DBP), with a statistically significant change between timepoints (Z = −2.98, n = 51, p = 0.030), with the median DBP 82 mmHg at baseline (IQR 77 mmHg to 92 mmHg), reducing to 79 mmHg at three-month follow-up (IQR 72 mmHg to 87 mmHg).

For further detail, see Supplementary Material S1 File.

## Discussion

By enabling the conceptualisation and visualisation of hypertension, this digital tool, when used within a pharmacy consultation, can improve patients’ perceptions of hypertension and perceived necessity of the medication. Preliminary observations also support the potential for the Hi-BP to improve hypertension control. It was feasible and acceptable for a researcher to deliver the Hi-BP intervention in a CP setting, from both patient and pharmacy staff perspectives. However, using pharmacists to deliver the intervention in Phase 2 was only partially acceptable, due to competing priorities and the current remuneration framework in Wales.

A strength of this work is derived from the multidisciplinary nature of the research team. The initial Hi-BP prototype included visual elements which were conceptualised in collaboration with art and design specialists, drawing on expertise from cardiovascular physiology, health psychology, pharmacy, data science and computer game design. Subsequent iterations of the Hi-BP intervention were co-produced and informed by patients, other stakeholders and members of the research team. Rousseau et al (2019) identified six intervention design approaches, with approaches involving stakeholders from multiple disciplines showing positive benefits for implementation arising from the diverse experiences within the team [46]. A further strength of this research was the adoption of the MRC framework for complex interventions [28] to guide intervention development and ensuring that the Hi-BP was theoretically based [18,47]. A robust exploration of intervention acceptability was guided by the use of the Theoretical Framework of Acceptability [44].

Patients and pharmacists provided positive feedback regarding Affective Attitude, i.e., how they felt about the intervention, its coherence and perceived effectiveness. Patients found Hi-BP interesting, providing reassurance to those who were already fully adherent to their prescribed anti-hypertensive treatment. Pharmacists felt Hi-BP was a useful tool and were keen to support the delivery of the intervention. Both patients and pharmacists understood the aim and purpose of Hi-BP. They believed the intervention strengthened understanding of hypertension and could act as a prompt to improving medication-taking behaviour. However, pharmacists taking part in Phase 2 of the study expressed frustration that they had not found the opportunity to engage with the Hi-BP intervention as planned. The lack of time to support medication adherence has been identified in the literature, both in the UK [36] and internationally [48,49]. A study of healthcare professional-led support for antiretroviral medication adherence identified a significant gap between the support the practitioners wished to provide versus the support they provided in reality, with lack of time identified as the main barrier [48].

There was strong support from patients for the fit of the intervention within a CP setting believing it to be more accessible than other healthcare settings. This is in line with previous UK-based studies exploring the accessibility of CP [50–52]. Positive attitudes regarding the suitability of CP for hypertension management have also been shown elsewhere [53,54]. Patients found the accessible language and visuals were a strength of the intervention, while noting the importance of an intervention facilitator with effective communication skills and sufficient subject knowledge. The approachability of pharmacy staff and their communication style has been noted as a key facilitator for participation in CP screening and minor ailment services [55,56]. Furthermore, research has shown that adherence interventions can be successfully delivered by members of the wider healthcare workforce, including community health workers and pharmacy technicians (PTs) [57,58] and that PTs have expressed willingness to expand their roles [59]. In Wales, some CP-based services are delivered by both pharmacists and PTs [60,61].

Findings from Phase 1 demonstrated that it is feasible to recruit to and facilitate the Hi-BP intervention in a CP setting, with no pharmacists reporting difficulties with recruitment. Pharmacists felt that providing support for medicines management was a core part of their role, suggesting that they also felt there was a place for adherence support interventions like Hi-BP within CP. However, they noted that opportunities to support adherence and medicines optimisation through formal remunerated services had decreased in the UK in recent years, as the CP Medicine Use Review Enhanced Service was discontinued in 2021 in both England and Wales [62,63]. However, CP-based medication adherence interventions have been shown to be effective [64,65], suggesting that CP is a suitable setting. In Phase 2, the patient recruitment target was not met, and pharmacists frequently mentioned time and workload pressure as barriers to patient recruitment and intervention delivery, with some actively recruiting patients only when they believed they could deliver the intervention. A systematic review exploring the implementation of pharmacy services suggested that scheduling appointments was helpful for both patients and pharmacists, although offering flexibility through allowing patients to ‘walk-in’ or prearrange an appointment was likely to be most beneficial for intervention implementation [66]. This aligns with our findings in terms of patient recruitment, with only 7% (5/69) of participants accessing Hi-BP without an appointment.

The outcome measures used in the preliminary testing of Hi-BP were found to be suitable, with no missing data in the self-report questionnaires. Whilst use of the MARS-5 in this population showed little variation in adherence scores, with a maximum possible median score demonstrated. This indicates a ‘ceiling effect’ of the measures due to a high proportion of participants reporting maximal adherence at baseline. Although scores in medication adherence did not change significantly over time, the range of scores did decrease, which may explain the lack of relationship between adherence and reduction in blood pressure. The limitations of adherence measures are well documented in the literature [67,68], with self-report measures considered susceptible to memory and desirability bias [69]. However, it is also accepted that there is no ‘gold standard’ measure, as all have strengths and weaknesses, [70]. Furthermore, our self-reported adherence results were supported by both prescription dispensing and pharmacy medication collection data.

When considering the limitations of this study, it should be noted that the Hi-BP intervention addressed non-adherence, however, this was not a recruitment criterion. Literature shows that the failure to target nonadherent participants in adherence studies is common, despite potentially contributing to the small effect sizes generally seen for such interventions [71]. This may have contributed to the potential ceiling effect seen with adherence outcomes. Furthermore, the patient participants were predominantly white; therefore the views shared and outcomes captured may not be reflective of the wider population. In addition, the pharmacists were all recruited from one CP group in one area of South-East Wales, potentially leading to homogenous views. For example, facilitators and barriers to intervention delivery may be related to characteristics of the pharmacy which may not be generalisable to other pharmacy chains. The study was conducted shortly after the UK emerged from a period of government-mandated social isolation during the COVID-19 pandemic, which may have influenced the working environment and context of the research.

Pharmacist views regarding acceptability were explored in both Phase 1 and 2. Semi-structured interviews were used in Phase 2, however, the pharmacist feedback from Phase 1 was provided via email. Semi-structured interviews gave the opportunity to explore comments in depth, whereas the written question-and-answer format made further exploration more difficult. Despite this, the feedback was similar overall, suggesting that the pharmacists had adequate opportunity to share their experience through either modality. Furthermore, the written format may have provided more opportunity for reflection before responding, therefore enabling the pharmacists to fully consider their experience of Hi-BP. The benefit of time for reflection has been identified as a strength of email interviews, being considered particularly beneficial where participants are being asked to recall past experiences as the asynchronous nature allows time for consideration and memory checking by the interviewee [72,73].

### Implications for practice and future research

While a larger evaluative trial is needed to explore the efficacy of Hi-BP in practice, this study highlights some important implications before this intervention can be successfully implemented in a CP setting. Having the optimal pharmacy team skill mix in place is important, since delegation of competing tasks to an appropriately qualified staff member may facilitate delivery. Appropriate remuneration is needed to facilitate the upskilling of staff to enable the delegation of more pharmacist duties. Similarly, in future studies, consideration should be given to the intervention facilitator. Widening the scope to include pharmacy technicians could increase facilitator availability and subsequent intervention delivery.

Further research is needed to identify the type of patients who will benefit most from the Hi-BP intervention and to develop a recruitment strategy to target patients whose BP is not controlled or are non-adherent to their antihypertensive treatment. In this study suggestions were made for its use with individuals newly diagnosed with hypertension, those managing multiple antihypertensives and for re-engaging individuals with a longstanding hypertension diagnosis. There is also the potential to integrate the intervention into other pharmacy services such as the Hypertension Case Finding Service [74] and New Medicines Services [75] which currently only exist in England. Research is also needed to explore the potential use HI-BP in primary prevention programmes for CVD such as screening services [76] and in different healthcare settings such as GP surgeries [77–79] or in secondary care clinics [80].

## Conclusions

This study found Hi-BP was feasible and acceptable to patients and pharmacists as a researcher-facilitated intervention in a CP setting. Both groups provided positive feedback about Hi-BP and patients liked the location due to its ease of access in the community. The outcome measures were appropriate for capturing illness perceptions, medication beliefs and antihypertensive adherence. Preliminary findings suggest that the Hi-BP intervention has the potential to make a major contribution to the secondary prevention of cardiovascular disease with significant reductions in both systolic and diastolic blood pressure at 3-months. However, this was a feasibility study which was not intended to determine the efficacy of the intervention and as such was not powered to do so. These feasibility findings will inform the design of a larger evaluative trial, with consideration given to enabling intervention delivery by both pharmacists and pharmacy technicians to remove identified barriers to intervention delivery and reflect the expansion of pharmacy services in the UK.

## Supporting information

S1 File Supplementary Material S1. S1 Table 1: Change in scores over time for B-IPQ constructs (n=54). S1 Table 2: Comparison of distribution of BMQ scores at baseline, T1 and T3 (n=54). S1 Table 3: MARS-5 scores across three time points (baseline, T1 immediately post-intervention and T3 three months post-intervention. S1 Table 4: Pharmacy data and blood pressure outcome measures at baseline and three-month follow-up. S1 Figure 1: Total MARS-5 scores at baseline, two-weeks post-intervention and 3-month follow-up (n=45). S1 Figure 2: Total recent adherence scores at baseline, 2-weeks post-intervention and 3-month follow-up (n=45)
(DOCX)

S2 File Supplementary Material S2. S2 Table 1: Qualitative thematic analysis of patient and pharmacist feedback with indicative quotes
(DOCX)
