# Supplementary material for: Visualisation of hypertension: A non-randomised pilot study to explore the feasibility of a Community Pharmacy-based intervention to support medication adherence (Hi-BP)
Source: PLoS One. 2026 Jan 2;21(1):e0339871. doi: 10.1371/journal.pone.0339871 (PMC12758789; doi:10.1371/journal.pone.0339871)
Supplement: S1 File — S1 Table 1: Change in scores over time for B-IPQ constructs (n=54). S1 Table 2: Comparison of distribution of BMQ scores at baseline, T1 and T3 (n=54). S1 Table 3: MARS-5 scores across three time points (baseline, T1 immediately post-intervention and T3 three months post-intervention. S1 Table 4: Pharmacy data and blood pressure outcome measures at baseline and three-month follow-up. S1 Figure 1: Total MARS-5 scores at baseline, two-weeks post-intervention and 3-month follow-up (n=45). S1 Figure 2: Total recent adherence scores at baseline, 2-weeks post-intervention and 3-month follow-up (n=45) (DOCX) [file pone.0339871.s001.docx]

Supplementary Material S1

1: Intervention overview and facilitator training

**Intervention overview**

Patients were recruited to the studies by the participating pharmacy teams and were invited to attend two consultations three months apart. The intervention was delivered during consultation one: quantitative outcome measures were recorded at baseline and immediately post-intervention. Follow-up outcome measures were collected in consultation two. The visual was web-based and displayed on a laptop computer in Phase 1. To avoid compatibility and security issues with pharmacy systems, the visual was accessed as a digital application using an electronic tablet (Lenovo™ android M10 FHD Plus) in Phase 2.

The intervention used the visual model and spoken narrative to convey six key messages:

1. The body silhouette and blood pressure monitor: What is blood pressure?
2. The artery cross-section: What is happening inside the artery?
3. The blood pressure scales: Comparing measured BP to ‘target’ BP
4. The artery cross-section: Comparing high and ideal BP
5. The stroke animation: What happens when a stroke occurs?
6. The role of medication: How does antihypertensive medication work?

The terms systolic and diastolic BP were explained verbally and visually. Patients were encouraged to compare the visualisation of a high and ideal BP and identify physiological changes found in a hypertensive artery. The Hi-BP intervention was tailored to the patient by measuring their BP and illustrating it visually. The mode of action of their antihypertensive medication was discussed using the identified physiological changes as a point of reference. The language used throughout the consultation was intentionally jargon-free where possible. Where technical terms were used (i.e., systolic and diastolic BP) these were also illustrated visually, and patient understanding was checked. The consultation broadly followed the format of the Medicine-Related Consultation Framework [1], used as a model of good practice in pharmacy consultations. A suggested narrative was provided for facilitators to encourage consistency of information; however, the conversation was not scripted to ensure natural delivery, encourage rapport between the patient and the facilitator and enable the information provided to be tailored to the needs of the individual.

**Facilitator training**

No facilitator training was necessary for Phase 1, as a member of the research team familiar with the intervention and consultation structure acted as facilitator. The researcher facilitator was a trained community pharmacist with the same underpinning hypertension knowledge as the pharmacist participants. Pharmacists facilitating the intervention in Phase 2 were accredited to provide community pharmacy enhanced services and as such had completed the Welsh National Enhanced Service Accreditation [2]. Pharmacists holding this qualification have demonstrated competence in communication and consultation skills.

Phase 1 pharmacists were trained in recruitment procedures, given a demonstration of the intervention and discussed the rationale for the project and psychological theories underpinning the intervention. In addition, pharmacists participating in Phase 2 had training on the intervention delivery. This covered the project background, refreshed knowledge of how hypertension affects the arteries, provided a clear consultation structure, and demonstrated how the visual model complemented the spoken narrative and supported the use of Hi-BP within a patient-centred consultation. This training was in the form of asynchronously viewed videos, written material and a 2-hour group workshop. Facilitators were given a printed consultation prompt and an intervention handbook as aide memoires.

2. Quantitative outcomes for Phase 1

S1 Table1 : Change in scores over time for B-IPQ constructs (n=54)

| **B-IPQ construct** | **Median (Baseline)**  **(inter-quartile range)** | **Median**  **(T1)**  **(inter-quartile range)** | **Median**  **(T3)**  **(inter-quartile range)** | **c^2^** | **Degrees of Freedom** | **Asymptotic Sig. (2-sided test)** |
| --- | --- | --- | --- | --- | --- | --- |
| Total B-IPQ | 32.50  (24.75-39.00) | 28.50  (24.00-35.50) | 30.00  (25.25-35.00) | 10.83 | 2 | **0.004** |
| Consequences | 1.00  (1.00 – 3.25) | 2.00  (1.00-4.25) | 2.00  (1.00-4.00) | 4.41 | 2 | 0.110 |
| Timeline | 10.00  (8.00-10.00) | 10.00  (7.00-10.00) | 10.00  (8.00-10.00) | 2.74 | 2 | 0.254 |
| Personal control | 7.00  (5.00-8.00) | 8.00  (5.00-9.00) | 7.00  (6.00-9.00) | 6.16 | 2 | **0.046** |
| Treatment control | 9.00  (7.00-10.00) | 9.00  (8.00-10.00) | 9.50  (8.00-10.00) | 9.04 | 2 | **0.011** |
| Identity | 2.00  (1.00-4.00) | 2.00  (1.00-4.00) | 2.00  (1.00-4.25) | 4.89 | 2 | 0.087 |
| Illness concern | 5.00  (1.00-7.25) | 5.00  (1.75-7.00) | 4.50  (1.75-6.25) | 0.78 | 2 | 0.679 |
| Illness coherence | 6.00  (4.75-9.00) | 9.00  (8.00-10.00) | 9.00  (7.75-10.00) | 32.35 | 2 | **<0.001** |
| Emotional representation | 1.00  (1.00-3.00) | 2.00  (1.00-4.00) | 2.00  (1.00-4.00) | 3.85 | 2 | 0.146 |

Emboldened values denote statistical significance (p=<0.05).

S1 Table 2: Comparison of distribution of BMQ scores at baseline, T1 and T3 (n=54)

| **BMQ construct** | **Median (Baseline)**  **(inter-quartile range)** | **Median (T1)**  **(inter-quartile range)** | **Median**  **(T3)**  **(inter-quartile range)** | **c^2^** | **Degrees of Freedom** | **Asymptotic Sig. (2-sided test)** |
| --- | --- | --- | --- | --- | --- | --- |
| Total BMQ necessity subscale | 17.50  (14.75 – 20.25 | 19.50  (17.00 – 21.00) | 19.00  (16.75– 20.25) | 11.71 | 2 | **.003** |
| Total BMQ Concern subscale | 11.50  (10.00 – 14.25) | 10.00  (8.00 – 12.25) | 11.00  (10.00– 13.00) | 9.95 | 2 | **.007** |
| Necessity-concern differential | 6.00  (2.00-  8.25) | 8.00  (4.75-11.25) | 7.00  (4.00-10.00) | 14.36 | 2 | **<.001** |

Emboldened values denote statistical significance (p=<0.05).


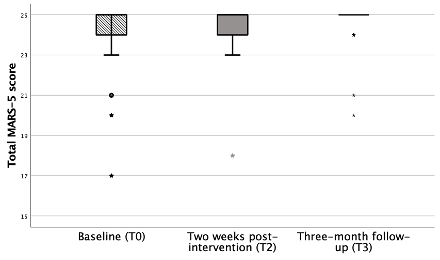


S1 Figure1: Total MARS-5 scores at baseline, two-weeks post-intervention and 3-month follow-up (n=45)


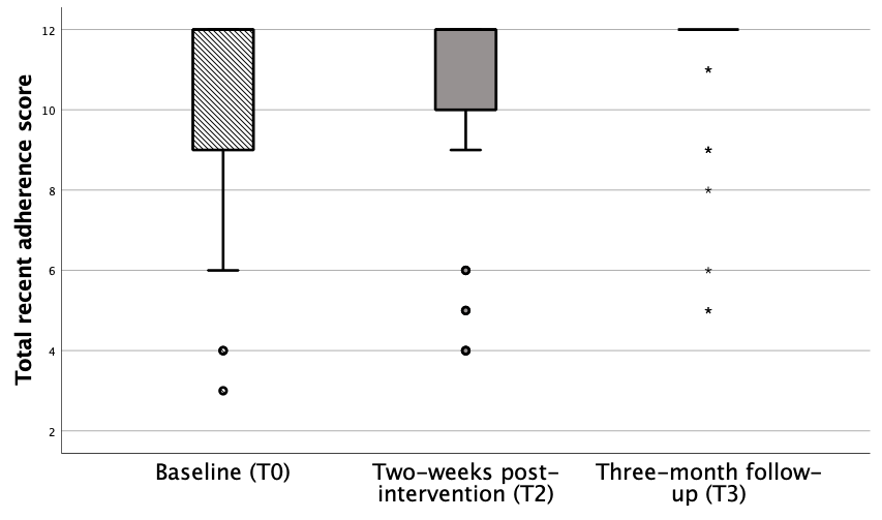


S1 Figure 2: Total recent adherence scores at baseline, 2-weeks post-intervention and 3-month follow-up (n=45)

S1 Table 3: MARS-5 scores across three time points (baseline, T1 immediately post-intervention and T3 three months post-intervention

| **Outcome** | **Median (Baseline)**  **(inter-quartile range)** | **Median (T1)**  **(inter-quartile range)** | **Median**  **(T3)**  **(inter-quartile range)** | **n** | **c^2^** | **Degrees of Freedom** | **Asymptotic Sig. (2-sided test)** |
| --- | --- | --- | --- | --- | --- | --- | --- |
| MARS-5 | 25.00  (24.00-25.00) | 25.00  (24.00-25.00) | 25 .00  (25.00-25.00) | 45 | 2.41 | 2 | 0.299 |
| Recent adherence | 12.00  (6.00 – 12.00) | 12.00  (9.00 – 12.00) | 12.00  (12.00 – 12.00) | 45 | 5.89 | 2 | 0.053 |

S1 Table 4: Pharmacy data and blood pressure outcome measures at baseline and three-month follow-up

| **Outcome** | **Median (Baseline)**  **(inter-quartile range)** | **Median**  **(T3)**  **(inter-quartile range)** | **n** | **Z** | **p** |
| --- | --- | --- | --- | --- | --- |
| vMPR (dispensing) | 1.00  (0.94 – 1.01) | 0.99  (0.92 - 1.00) | 68 | -0.76 | 0.455 |
| vMPR (collection) | 1.00  (0.94 – 1.01) | 0.99  (0.92 – 1.00) | 45 | -1.10 | 0.271 |
| Systolic blood pressure | 148mHg  (135mmHg – 160mmHg) | 133mmHg  (126mmHg – 148mmHg) | 51 | -3.91 | <0.001 |
| Diastolic blood pressure | 82mmHg  (77mmHg – 92mmHg) | 79mmHg  (72mmHg – 87mmHg) | 51 | -2.98 | 0.030 |

References

1. Abdel-Tawab R, James DH, Fichtinger A, Clatworthy J, Horne R, Davies G. Development and validation of the Medication-Related Consultation Framework (MRCF). Patient Educ Couns. 2011;83: 451–457. doi:10.1016/j.pec.2011.05.005

2. HEIW. Pharmacy Enhanced Services. [cited 1 Jul 2021]. Available: https://www.wcppe.org.uk/enhanced-services/
